# Uncovering the genetic relationship between dementia and age‐related hearing loss

**DOI:** 10.1002/alz70855_098698

**Published:** 2025-12-23

**Authors:** Andrew Van Domelen, Samah Ahmed, Kenneth I Vaden, Judy R Dubno, Britt Drögemöller

**Affiliations:** ^1^ University of Manitoba, Winnipeg, MB, Canada; ^2^ Medical University of South Carolina, Charleston, SC, USA; ^3^ The Children's Hospital Foundation of Manitoba, Winnipeg, MB, Canada; ^4^ CancerCare Manitoba Research Institute, Winnipeg, MB, Canada; ^5^ Centre on Aging, Winnipeg, MB, Canada

## Abstract

**Background:**

Hearing loss is an important modifiable midlife risk factor for dementia. Recent studies have explored a potential genetic relationship between dementia and age‐related hearing loss (ARHL). However, due to variability in phenotype definitions and analysis methods, these studies have not reached a clear consensus on the specific genetic factors underlying both conditions. We have previously investigated the genetics of different ARHL subtypes and have shown that genes linked to frontotemporal dementia also play a role in the metabolic subtype of ARHL. By employing a comprehensive phenotyping strategy that involves highly specific, objective measures of cognition and audiological measures of hearing, we aim to examine the genetic relationship between dementia and ARHL with increased granularity.

**Method:**

Through the Canadian Longitudinal Study on Aging (CLSA), we have access to high‐quality genotype, cognitive, and audiological data for 17,221 older adults. Estimates of hearing loss subtypes were calculated from CLSA participant audiograms. Cognitive impairment, an early dementia trait, was defined using a CLSA‐specific indicator and the association between this indicator and hearing loss subtypes was investigated. In the coming months, we aim to assess potential causality between cognitive impairment and the metabolic subtype of ARHL using Mendelian randomization. We will also apply regression and fine‐mapping analyses to identify genetic variants, genes and pathways that may affect both dementia and ARHL.

**Result:**

Investigation of the relationship between cognitive impairment and ARHL subtypes identified a highly significant association with estimates of metabolic hearing loss (*p* = 3x10^‐9^; OR[95%CI]=1.016[1.011,1.022]), whereas no significant association was observed with sensory hearing loss (*p* = 0.35; OR[95%CI]=0.99[0.988,1.004]). We are currently preparing genetic data for further analysis and expect to uncover genetic variants that correlate with both metabolic hearing loss and cognitive impairment and/or establish a causative pathway between these phenotypes.

**Conclusion:**

This study has shown cognitive impairment to be significantly associated with the metabolic subtype of ARHL. Identifying correlated genetic variants and/or a causal link between these conditions will enhance our understanding of the biological mechanisms underlying the genetics of dementia and ARHL. Additionally, our findings may guide the development of improved dementia prediction tools through the incorporation of measures of hearing and genetic data.

